# Senotherapeutic Peptide 14 Suppresses Th1 and M1 Human T Cell and Monocyte Subsets In Vitro

**DOI:** 10.3390/cells13100813

**Published:** 2024-05-10

**Authors:** Thuany Alencar-Silva, Stefhani Martins de Barcelos, Amandda Silva-Carvalho, Mauricio Gonçalves da Costa Sousa, Taia Maria Berto Rezende, Robert Pogue, Felipe Saldanha-Araújo, Octávio Luiz Franco, Mariana Boroni, Alessandra Zonari, Juliana Lott Carvalho

**Affiliations:** 1 Post-Graduation Program in Genomic Sciences and Biotechnology, Catholic University of Brasília, Brasília 71966-700, Brazilstefhanibarcelos16@gmail.com (S.M.d.B.); sousam@ohsu.edu (M.G.d.C.S.); taiambr@gmail.com (T.M.B.R.); redward@p.ucb.br (R.P.);; 2 Multidisciplinary Laboratory of Biosciences, Faculty of Medicine, University of Brasília, Brasília 70910-900, Brazil; 3 Hematology and Stem Cell Laboratory, Faculty of Health Sciences, University of Brasília, Brasília 70910-900, Brazil; amanddaevelinsc@gmail.com (A.S.-C.);; 4 Dentistry Department, University of Brasília, Brasília 70910-900, Brazil; 5 Post-Graduation Program in Health Sciences, University of Brasília, Brasília 70910-900, Brazil; 6 Centre of Proteomic Analyses and Biochemistry, Genomic Sciences and Biotechnology Program, Catholic University of Brasília, Brasília 71966-700, Brazil; 7 S-Inova Biotech, Biotechnology Program, Catholic University Dom Bosco, Campo Grande 79117-900, Brazil; 8 Molecular Pathology Program, University of Brasília, Brasília 70910-900, Brazil; 9 OneSkin, Inc., San Francisco, CA 94107, USA; 10 Bioinformatics and Computational Biology Lab, Brazilian National Cancer Institute (INCA), Rio de Janeiro 20230-130, Brazil

**Keywords:** multifunctional peptides, immunomodulation, inflammaging, aging

## Abstract

Inflammation contributes to the onset and exacerbation of numerous age-related diseases, often manifesting as a chronic condition during aging. Given that cellular senescence fosters local and systemic inflammation, senotherapeutic interventions could potentially aid in managing or even reducing inflammation. Here, we investigated the immunomodulatory effects of the senotherapeutic Peptide 14 (Pep 14) in human peripheral blood mononuclear cells (PBMCs), monocytes, and macrophages. We found that, despite failing to significantly influence T cell activation and proliferation, the peptide promoted a Th2/Treg gene expression and cytokine signature in PBMCs, characterized by increased expression of the transcription factors *GATA3* and *FOXP3*, as well as the cytokines IL-4 and IL-10. These observations were partially confirmed through ELISA, in which we observed increased IL-10 release by resting and PHA-stimulated PBMCs. In monocytes from the U-937 cell line, Pep 14 induced apoptosis in lipopolysaccharide (LPS)-stimulated cells and upregulated *IL-10* expression. Furthermore, Pep 14 prevented LPS-induced activation and promoted an M2-like polarization in U-937-derived macrophages, evidenced by decreased expression of M1 markers and increased expression of M2 markers. We also showed that the conditioned media from Pep 14-treated macrophages enhanced fibroblast migration, indicative of a functional M2 phenotype. Taken together, our findings suggest that Pep 14 modulates immune cell function towards an anti-inflammatory and regenerative phenotype, highlighting its potential as a therapeutic intervention to alleviate immunosenescence-associated dysregulation.

## 1. Introduction

The effects of aging on the immune system, also known as immunosenescence [1], contribute to the occurrence of a chronic state in which excessive inflammatory signaling and exhaustion of adaptive immunity are observed. At the cellular level, immunosenescence is generally defined by the reduction in peripheral blood naïve CD8+ T cells combined with the accumulation of late-stage differentiated and senescent immune cells [2], though significant alterations in innate immune cells have also been documented [3]. At the intercellular communication level, immunosenescence is defined by excessive inflammatory signaling, reflected in elevated circulating IL-6 levels [4], and compromised Treg function [5]. Clinically, increased basal levels of inflammatory mediators are documented, even in the absence of infection [6]. This results in a compromised ability of the immune system to fight infections, a greater prevalence of autoimmune and inflammatory disorders, and the aggravation of degenerative diseases with advanced age.

Currently, there is a lot of interest in the development of therapies that limit immunosenescence and rejuvenate the immune system. In this context, senotherapeutic molecules are known to reduce the burden of cellular senescence, constituting a promising therapeutic approach to reduce the accumulation of senescent cells observed in aging as well as in age-related diseases [7].

Peptide 14 (Pep 14) is a synthetic peptide that was described by our group as a result of a phenotypic screening and optimization process performed to identify effective and non-toxic senotherapeutic molecules [8]. Using different in vitro models of dermal fibroblast senescence (e.g., primary cells derived from Hutchinson–Gilford progeria syndrome donors, chronological aging, ultraviolet-B radiation (UVB), and etoposide treatment), we showed that Pep 14 effectively modulates cellular senescence and inflammatory senescence-associated secretory phenotype (SASP) signaling. At the single-cell level, the peptide promoted the transcription of DNA repair genes in early-senescent cells, possibly preventing them from becoming late-senescent, high-SASP-producing cells. Ultimately, such alterations resulted in a significant reduction in the biological age of human skin, in vitro [8]. Currently, the peptide is being explored for dermatological purposes.

Mechanistically, Pep 14 appears to act by stabilizing the PP2A complex [8]. PP2A is a complex phosphatase comprising heterotrimeric Ser/Thr subunits that regulate several cellular processes. Current evidence suggests that PP2A plays a significant role in inflammatory responses. Despite not being extensively investigated in the context of autoimmunity, alterations of PP2A were associated with compromised Treg function and different inflammatory diseases, as recently reviewed [9]. Considering the role of PP2A in controlling the immune system, we hypothesized that the peptide might also exert direct effects on immune cells.

In the present study, we determined the effects of Pep 14 in human peripheral blood mononuclear cells (PBMCs), as well as in human monocytes and macrophages of the U-937 cell line. Considering that several age-related diseases involve inflammatory processes, the investigation of how immune cells respond to Pep 14 can offer valuable insights into the potential role of this peptide in modulating critical processes related to inflammation.

## 2. Materials and Methods

### 2.1. Peptide Synthesis

Pep 14 (ETAKHWLKGI) was synthesized by CPC Scientific (Sunnyvale, CA, USA) and reconstituted in ultrapure water. The peptide was aliquoted and stored at −20 °C until use.

### 2.2. Isolation and Culture of Peripheral Blood Mononuclear Cells (PBMCs)

PBMCs were obtained from healthy volunteers by centrifugation using Ficoll-Paque PLUS (Amersham Biosciences, Uppsala, Sweden), as approved by the Ethics Committee of the Faculty of Medicine of the University of Brasília (protocol 35640512.5.0000.0030). After isolation, PBMCs were either left in a resting state or treated with a stimulating agent (5 μg/mL phytohemagglutinin—PHA) and treated or not with Pep 14 (3.12 µM or at a different concentration, as stated in each experiment) or rapamycin (100 nM). Then, cells were cultured under different stimuli for 24 h (CD69 staining) or 48 h (viability assay) in RPMI 1640 medium (Gibco, Waltham, MA, USA) supplemented with 10% v.v. fetal bovine serum (FBS) (Gibco, USA), in addition to 1% v.v. penicillin/streptomycin solution (1000 U/mL) (Invitrogen, Grand Island, NY, USA), and maintained at 5% CO_2_, 37 °C, and 95% humidity.

### 2.3. PBMC Viability Assay

The toxicity of Pep 14 on PBMCs was evaluated using the 3-[4,5-dimethylthiazol-2-yl]-2,5-diphenyltetrazolium bromide (MTT; Sigma Chemicals Co., St. Louis, MO, USA) metabolization assay. To do so, PBMCs were treated with different concentrations of the peptide for 48 h (1.56, 3.12, 6.25, 12.5, 25, 50, 100, and 150 µM). Then, the media were changed, and fresh media containing 5 mg/mL MTT solution were added to each well. After 4 h of protection from light, 100 µL of dimethyl sulfoxide (DMSO) was added to each well. Absorbance was then determined in each well using an ELISA reader at 570 and 630 nm. The experiment was performed as three independent replicates, and each experimental replicate was performed as technical duplicates. Data were normalized to untreated controls.

### 2.4. PBMC Proliferation Assay

Cell proliferation was assessed using the carboxyfluorescein succinimidyl ester (CFSE) assay, as previously published by our group [10]. To do so, PBMCs were labeled with 2.5 µM CFSE (Sigma-Aldrich, St. Louis, MO, USA), treated with Pep 14 (3.12 µM) or rapamycin (100 nM), and stimulated or not with PHA (5 μg/mL). On the fifth day after treatment (120 h), cells were harvested, labeled with anti-CD3-PerCP antibodies (eBioscience, San Diego, CA, USA), and evaluated by flow cytometry using a FACSCalibur (BD Biosciences, San Jose, CA, USA). Data were analyzed using FlowJo 10.0.7 software. Ten thousand events were recorded per sample. The experiment was performed using PBMCs isolated from four healthy donors.

### 2.5. T Cell Activation Assay

PBMCs were cultured and treated as previously described. After 24 h, cells were harvested and stained for the early T cell activation marker CD69 (anti-CD69-FITC) as well as anti-CD3-PerCP antibodies (eBioscience, San Diego, CA, USA) and analyzed by flow cytometry (FacsVerse, BD Bioscience, USA) using FlowJo 10.0.7 software. Ten thousand events were recorded per sample. The experiment was performed using PBMCs isolated from four healthy donors.

### 2.6. Human Monocyte Cell Cultures

Human monocyte cultures (U-937 cell line—Banco de Células do Rio de Janeiro, Brazil) were maintained in RPMI 1640 medium supplemented with 10% v.v FBS and 1% v.v. penicillin/streptomycin solution (1000 U/mL) and incubated at 5% CO_2_, 37 °C, and 95% humidity. For cell viability and NO quantification, cells were plated in 96-well plates and treated for 48 h.

### 2.7. U-937 Cell Viability Assay

U-937 cells were plated in 96-well plates and either left in a resting state or treated with 0.5 µg/mL LPS and/or 3.12 µM Pep 14 for 24 h. Then, the MTT assay was performed to evaluate the viability of cell cultures, as described above for PBMCs.

### 2.8. U-937 Macrophage Differentiation

U-937 cells were treated with phorbol ester (PMA) at a concentration of 25 ng/mL. After 72 h, the differentiation of U-937 cells into macrophages was observed, as described by PINTO et al. (2021) [11]. Non-differentiated cells (non-adherent) were removed by washing the plates with fresh media. For cell viability and NO quantification, cells were plated in 96-well plates and treated for 48 h; for qRT-PCR and supernatant collection for ELISA experiments, cells were plated in 6-well plates and treated for 24 h.

### 2.9. Nitric Oxide Release

Nitric oxide (NO) was measured in the U-937 and U-937-derived macrophage culture supernatants, as previously described [12]. Briefly, cells were plated and left undisturbed or treated with 0.5 µg/mL LPS and/or 3.12 µM Pep 14 for 48 h. Culture supernatants (100 μL) were transferred to 96-well plates (Kasvi, São José dos Pinhais, PR, Brazil), and 100 μL of supplemented RPMI medium was added to the wells of the sodium nitrite standard curve (Gibco, USA). Next, 100 μL of a 1% sulfanilamide solution was added to 2.5% phosphoric acid and 1% naphthyl ethylenediamine in 2.5% phosphoric acid at a 1:1 ratio. The reading was performed on a microplate reader (Bio-Tek PowerWave HT, Winooski, VT, USA) after 10 min of incubation at room temperature at a wavelength of 490 nm. From the standard curve for sodium nitrite (1.5625 μM to 200 μM), a calculation was performed to estimate the amount of nitrite detected in each sample.

### 2.10. Enzyme-Linked Immunosorbent Assay (ELISA)

The production of IL-10 and TNF-α cytokines was evaluated in the culture supernatant of PBMCs and macrophages treated for 24 h with LPS (0.5 µg/mL) and/or Pep 14 (3.12 µM), following the manufacturer’s specifications (Peprotech, Waltham, MA, USA). Cytokine levels were expressed in picograms per milliliter (pg/mL), as quantified according to the standard curves built following the manufacturer’s specifications.

### 2.11. Real-Time PCR

Total RNA from cells in each experimental condition was isolated using Trizol reagent (Gibco), according to the manufacturer’s instructions. RNA concentration was determined using a Nanodrop spectrophotometer (Thermo Fisher Scientific, Waltham, MA, USA). The RNA samples were subjected to reverse transcription using the High-Capacity cDNA Reverse Transcription Kit (Thermo Fisher Scientific, USA), following the manufacturer’s instructions. Then, qPCR reactions were prepared using the obtained cDNA samples, the SYBR™ Green Master Mix (Thermo Fisher Scientific, Waltham, MA USA), and specific primers for each gene (Table 1). Amplifications were conducted using StepOne Plus system (Applied Biosystems, Waltham, MA, USA), and data were analyzed using StepOne Software v2.3. The results are expressed as CT values. Individual CT readings were transposed into spreadsheets for 2^−ΔΔCT^ analysis, as proposed by NOLAN, HANDS, and BUSTIN in 2006 [13].

The mRNA expression of *GAPDH* (housekeeping), *FOXP3* (master transcription factor in regulating Treg cell development and function), *GATA3* (master regulator of Th2-cell differentiation), *TBET* (master regulator of Th1 cell development), *TGF-β*, *IL-10* (Treg effector cytokines), *IL-4* (Th2 effector cytokine), *TNF-α*, *IFN-γ* (Th1 effector cytokines), *NF-κB1*, *REL*, *RELA* (canonical NF-κB signaling pathway), *NF-κB2*, and *RELB* (non-canonical NF-κB signaling pathway) was determined for PBMCs of different experimental groups after 120 h treatments. Monocytes were subjected to expression analysis of *TNF-α*, and *IL-10* after 24 h treatments. Similarly, after 24 h treatments, macrophages were subjected to expression analysis of *GAPDH* (housekeeping); *IFN-γ*, *IL-1β*, and *TNF-α* (M1 polarization); *IL-10*, *IL-4*, *IL-1R1*, *ARG1*, and *TGF-β* (M2 polarization); *HLA-DQA1* (macrophage activation); and *GATA3*, *TBET*, *NF-κB1*, and *NF-κB2* (inflammation and macrophage polarization).

### 2.12. Cell Migration Assay

For the migration assay, 2 × 10^5^ fibroblasts were plated in 6-well plates in DMEM supplemented with 10% v.v. FBS and 1% v.v. penicillin/streptomycin solution (1000 U/mL) and kept at 5% CO_2_, 37 °C, and 95% humidity. When they reached confluence, a space was created between the cells (scratches) using a 200 µL volume pipette, as previously described [14]. After scratching, the medium was removed, the wells were washed with PBS, and fresh medium without FBS was added. Then, positive migration control wells received fresh media containing 2% FBS. As a negative control, cells were maintained in the absence of FBS and without experimental treatment. The experimental groups were maintained under serum-starved conditions and treated with 50% v.v. DMEM without FBS and 50% v.v. of conditioned media obtained from macrophages previously treated and not treated with Pep 14 (3.12 μM) and/or LPS (0.5 μg/mL) for 48 h. The plates containing the cells were incubated at 37 °C and photo-monitored at previously established intervals (0, 24, and 48 h). Subsequently, the number of cells that invaded the scratched area was counted using the ImageJ program, version 1.53 i (National Institutes of Health, Bethesda, Maryland, USA)and the cell migration capacity was measured for the groups exposed or not exposed to the peptide and FBS. The number of cells in the positive control group that migrated into the scratch area at 48 h was then considered to be 100% migration.

### 2.13. Statistical Analysis

Normality analysis was performed using the Shapiro–Wilk test. Statistical differences between groups were evaluated using analysis of variance (ANOVA), which was used to evaluate cell viability, migration, proliferation, and gene expression, followed by the post hoc Tukey test for multiple comparisons. Analyses were performed considering a 95% confidence interval and a *p*-value of 0.05. All statistical analyses were performed using GraphPad Prism^®^ software, version 7.02 (San Diego, CA, USA, 2017).

## 3. Results

### 3.1. Pep 14 Promotes Th2 and Treg mRNA and Cytokine Signature in Phytohemagglutinin-Treated PBMCs

**Figure 1:**
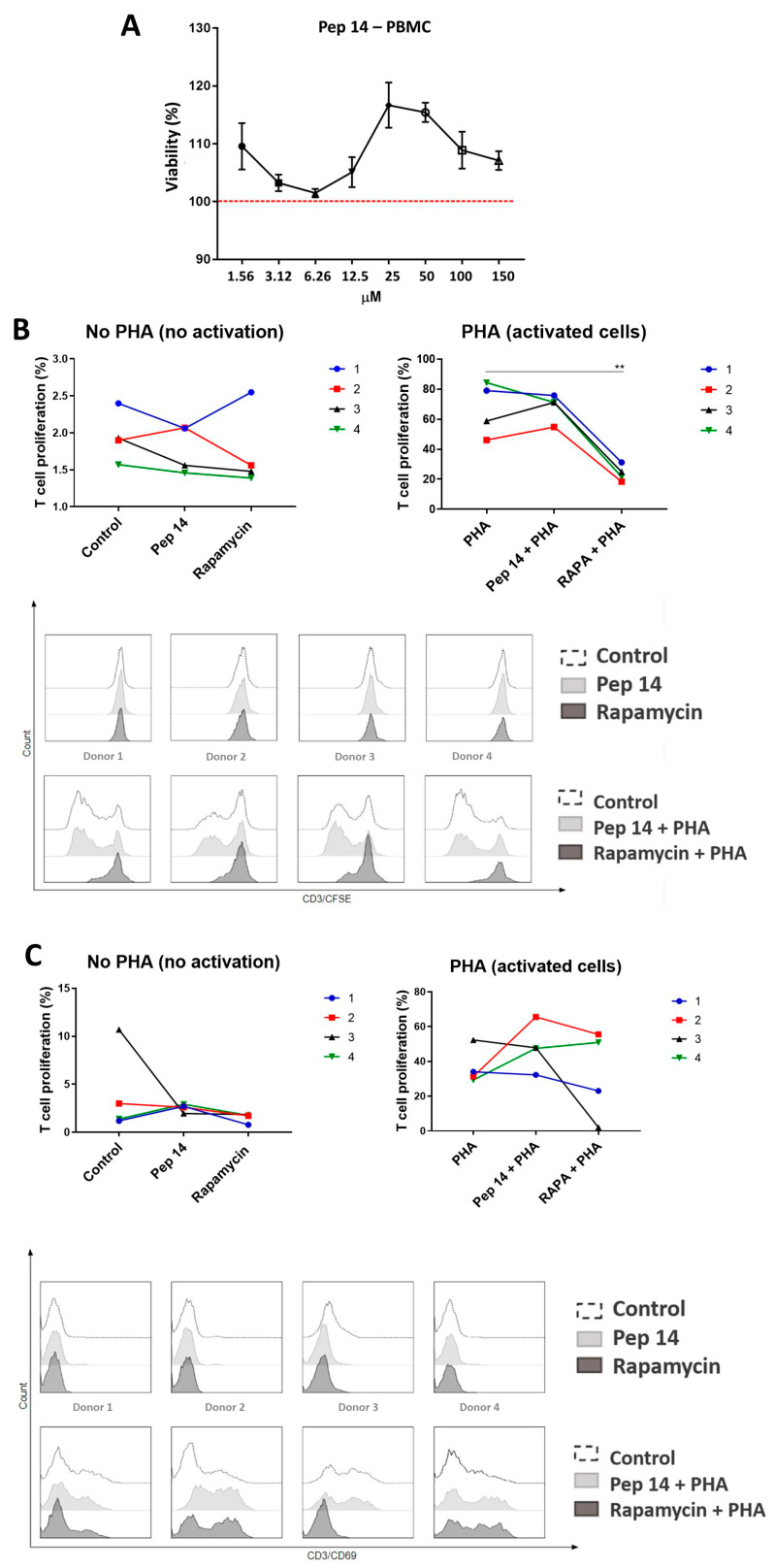
Viability and proliferation of PBMCs treated with Pep 14. (**A**) The cellular viability of PBMCs treated with various concentrations of Pep 14 was determined using the MTT assay. Data are presented as a percentage of positive viability control (non-treated PBMCs). (**B**) Proliferation of resting (no PHA) and activated (PHA-treated) T cells obtained from 4 donors (1–4) and treated with 3.12 μM Pep 14 or (100 nM) rapamycin. Cells were stained with CFSE and treated with PHA and/or Pep 14 for 120 h. Data are presented as a percentage of proliferating T cells relative to total cells. (**C**) Activation of resting and activated T cells treated with 3.12 μM Pep 14. Cells were treated with PHA and/or Pep 14 for 24 h and then labeled with anti-CD3 and anti-CD69 antibodies. Data are presented as mean ± SD of the percentage of activated T cells relative to total cells. The experiments were performed using four biological samples numbered 1–4. Statistical tests were performed using analysis of variance, followed by post hoc Tukey tests. ** *p* < 0.01 compared to the control group.

Human PBMCs from three donors were cultured for 24 h in the presence of increasing doses of Pep 14 (1.56–150 μM). Importantly, Pep 14 did not alter cell viability in a significant way (Figure 1A). After the determination of Pep 14 toxicity for PBMCs, a concentration of 3.12 μM was selected for subsequent experiments since it is the lowest concentration described as effective for both healthy and Hutchinson–Gilford progeria-derived human fibroblasts [8].

Then, we tested the effects of Pep 14 treatment on T cell proliferation and activation, two primary events for adaptive immune response-mediated inflammation. For this experiment, PHA was used as a T cell activation/proliferation stimulus, and rapamycin was used as a reference immunosuppressant molecule [15]. Our results showed that Pep 14 treatment alone failed to significantly promote T cell proliferation (Figure 1B) and activation (Figure 1C). Furthermore, the peptide also failed to significantly alter T cell activation after PHA stimulation. Rapamycin, on the other hand, used as a reference immunomodulatory molecule [15], significantly limited T cell proliferation, as expected for an immunosuppressant drug.

**Figure 2:**
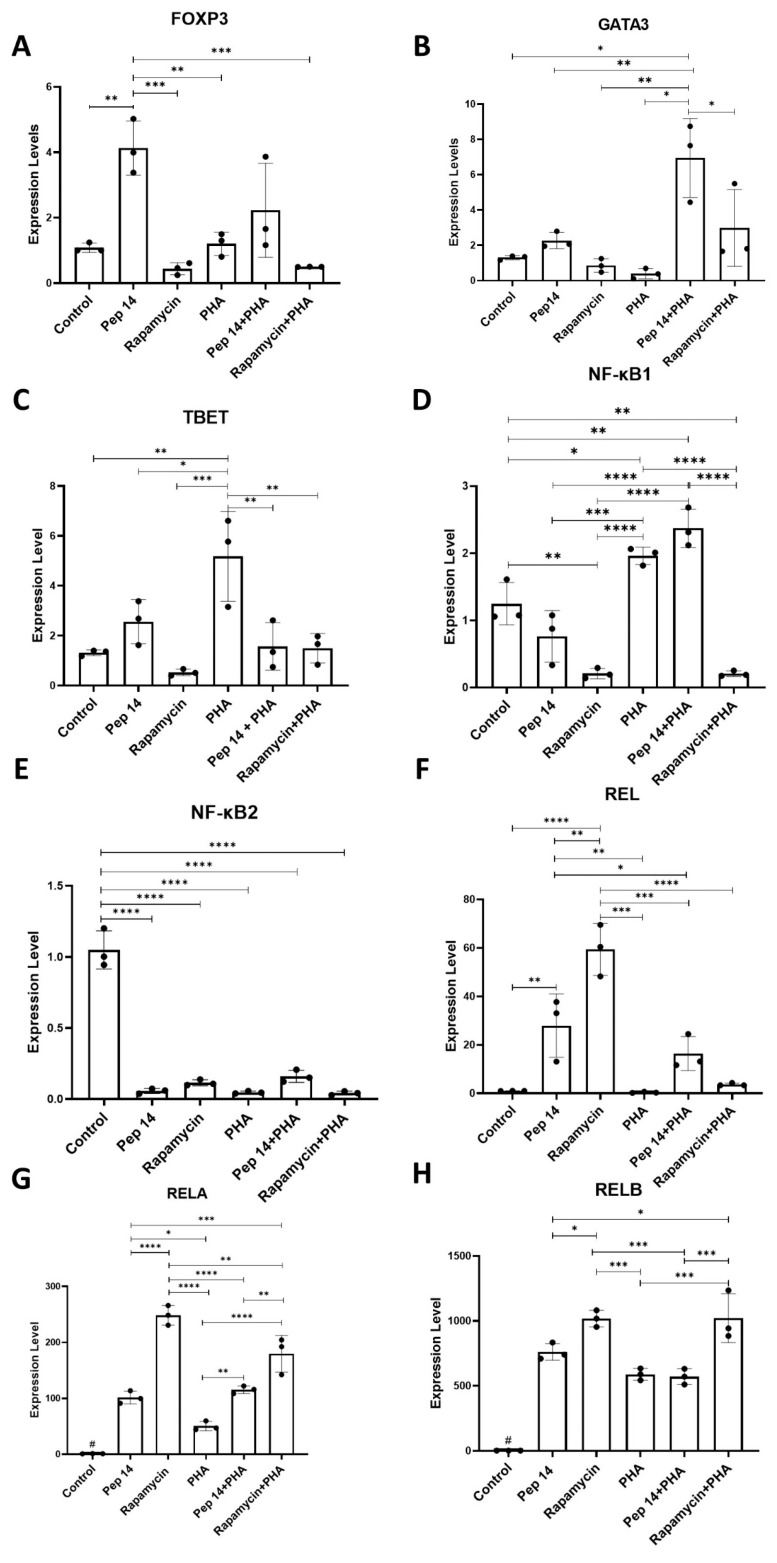
Comparative analysis of gene expression levels involved in T cell differentiation, including specific markers for Th1, Th2, and Treg effector lymphocytes. The samples were subjected to real-time PCR (qPCR) analysis to quantify the expression of (**A**) *FOXP3*, (**B**) *GATA3*, (**C**) *TBET*, (**D**) *NF-κB1*, (**E**) *NF-κB2*, (**F**) *REL*, (**G**) *RELA*, and (**H**) *RELB.* All data were normalized to the control group. Statistical analysis was performed using ANOVA, followed by Tukey’s post hoc test for multiple comparisons. * *p* < 0.05, ** *p* < 0.01, *** *p* < 0.001, **** *p* < 0.0001. # *p* < 0.05 between control and experimental groups.

To explore other possible effects of Pep14 on PBMCs during PHA stimulation, we analyzed the mRNA expression of relevant genes involved in T cell differentiation, namely *TBET*, a regulator in Th1 differentiation; *GATA3* a regulator in Th2 differentiation; and *FOXP3*, a determinant for Treg differentiation. Our results show that in resting PBMCs, Pep 14 promoted a significant increase in *FOXP3* mRNA levels, while not significantly influencing *GATA3* and *TBET* expression (Figure 2A–C), when compared to untreated controls. In PHA-stimulated PBMCs, the Pep 14-induced increase in *FOXP3* was more discrete. Furthermore, Pep 14 significantly promoted *GATA3* mRNA expression and abolished the PHA-induced increase in *TBET* mRNA levels, compared to the PHA-stimulated control. Rapamycin treatment also prevented PHA-induced *TBET* expression, and showed a trend towards increased *GATA3* levels, compared to the PHA-stimulated control.

Given that IL-10 and IL-4 are signature cytokines of Th2 cells, and that the *GATA3* gene was induced following Pep 14 stimulation, we investigated the mRNA levels of genes associated with T cell differentiation, including members of the NF-κB family *NF-κB1* (Figure 2D) and *NF-κB2* (Figure 2E), the transcriptional regulator REL (*c-REL*) (Figure 2F), and those involved in canonical and non-canonical activation pathways (*RELA* and *RELB*, respectively) (Figure 2G,H) *RELA* is necessary for Th1 differentiation, but its recruitment to the *IFN-γ* enhancer region is dependent on TBET expression in Th1 cells [16]. The RELB protein, primarily involved in the non-canonical activation of the NF-κB pathway, is important for Treg differentiation and function, with *RELB* defects being associated with autoimmune disorders [17].

Our results revealed that in resting PBMCs, *NF-κB2* expression was significantly reduced upon Pep 14 treatment (Figure 2E), while *RELA* and *RELB* (Figure 2G,H) mRNA levels were significantly increased. In PHA-stimulated PBMCs, Pep 14 promoted significantly increased *REL* and *RELA* (Figure 2F,G) mRNA levels. Rapamycin significantly reduced *NF-κB2* (Figure 2E) and significantly increased *REL*, *RELA*, and *RELB* (Figure 2F–H) levels in resting PBMCs. In activated counterparts, rapamycin significantly reduced *NF-κB1* (Figure 2D) and increased *REL* (Figure 2F) mRNA levels, indicating distinct mechanisms associated with Pep 14 and rapamycin.

**Figure 3:**
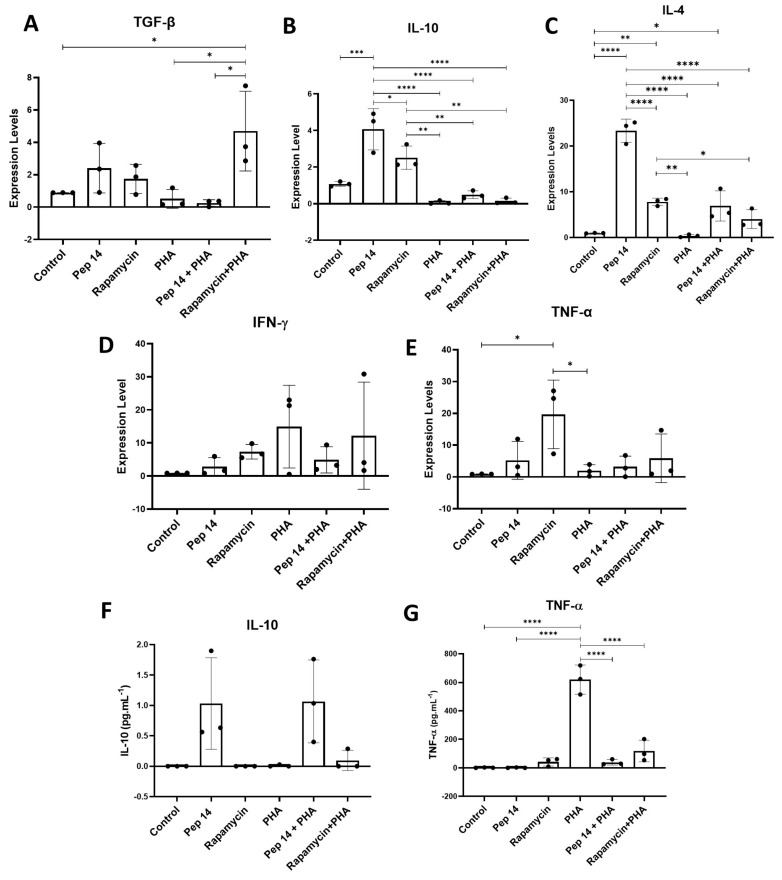
Comparative analysis of gene expression levels involved in T cell differentiation, including specific markers for Th1, Th2, and Treg effector lymphocytes. The expression levels of transcripts associated with Th1, Th2, and Treg effector lymphocytes were evaluated: (**A**) *TGF-β*, (**B**) *IL-10*, (**C**) *IL-4*, (**D**) *IFN-γ*, and (**E**) *TNF-α*. To validate the results obtained by qPCR, the expression levels of (**F**) IL-10 and (**G**) TNF-α proteins were quantified by enzyme-linked immunosorbent assay (ELISA). All data were normalized to the control group. Statistical analysis was performed using ANOVA, followed by Tukey’s post hoc test for multiple comparisons. * *p* < 0.05, ** *p* < 0.01, *** *p* < 0.001, **** *p* < 0.0001 between groups.

Considering the possible effect of Pep 14 in supporting the Treg and Th2 effector T cell subsets, we investigated the mRNA levels of cytokines associated with Th1, Th2, and Treg effector lymphocyte subsets. Using qRT-PCR, we evaluated the Th1-related transcripts *IFN-γ* and *TNF-α*, the Th2-related transcript *IL-4*, and *TGF-β* and *IL-10*, associated with Treg function and differentiation [18] (Figure 3). In resting PBMCs, Pep 14 treatment promoted a significant increase in *TGF-β*, *IL-10*, and *IL-4* expression (Figure 3A–C) compared to the untreated control. Compared to PHA-activated cells, PBMCs stimulated with both PHA and Pep 14 showed a trend towards increased *IL-10* and *IL-4* mRNA levels (Figure 3B,C), as well as a non-significant reduction in *IFN-γ* expression (Figure 3D). In resting PBMCs, rapamycin promoted a significant increase in *TNF-α* (Figure 3E), as well as a trend towards increased *IL-10* and *IL-4* (Figure 3B,C), compared to the untreated control. In PHA-activated PBMCs, rapamycin treatment promoted a significant increase in *TGF-β* transcript levels (Figure 3A) compared to the PHA-stimulated control. Given that IL-10 and IL-4 are signature cytokines of Th2 cells, our findings corroborate the notion that Pep 14 treatment supports Th2 and Treg effector T cell subsets.

As a validation of the qRT-PCR results, IL-10 and TNF-α protein levels were determined using ELISA (Figure 3F,G). Once again, IL-10 levels were significantly increased following Pep 14 treatment, both in resting and PHA-treated PBMCs, compared to the respective untreated and PHA-stimulated controls. Different from the qRT-PCR findings, rapamycin treatment failed to promote IL-10 release in the supernatant of resting PBMCs. For TNF-α, no significant influence of Pep 14 and rapamycin was observed in the supernatant of resting PBMCs. Nevertheless, in PHA-treated PBMCs, both Pep 14 and rapamycin treatments prevented a PHA-induced increase in TNF-α. Together, our findings suggest that Pep 14 modulates T cells in vitro by suppressing the Th1 phenotype and cytokine release, therefore favoring the Treg and Th2 phenotypes.

### 3.2. Pep 14 Influences LPS-Stimulated U-937 Monocytes by Inducing Apoptosis of Activated Cells and IL-10 Expression

**Figure 4:**
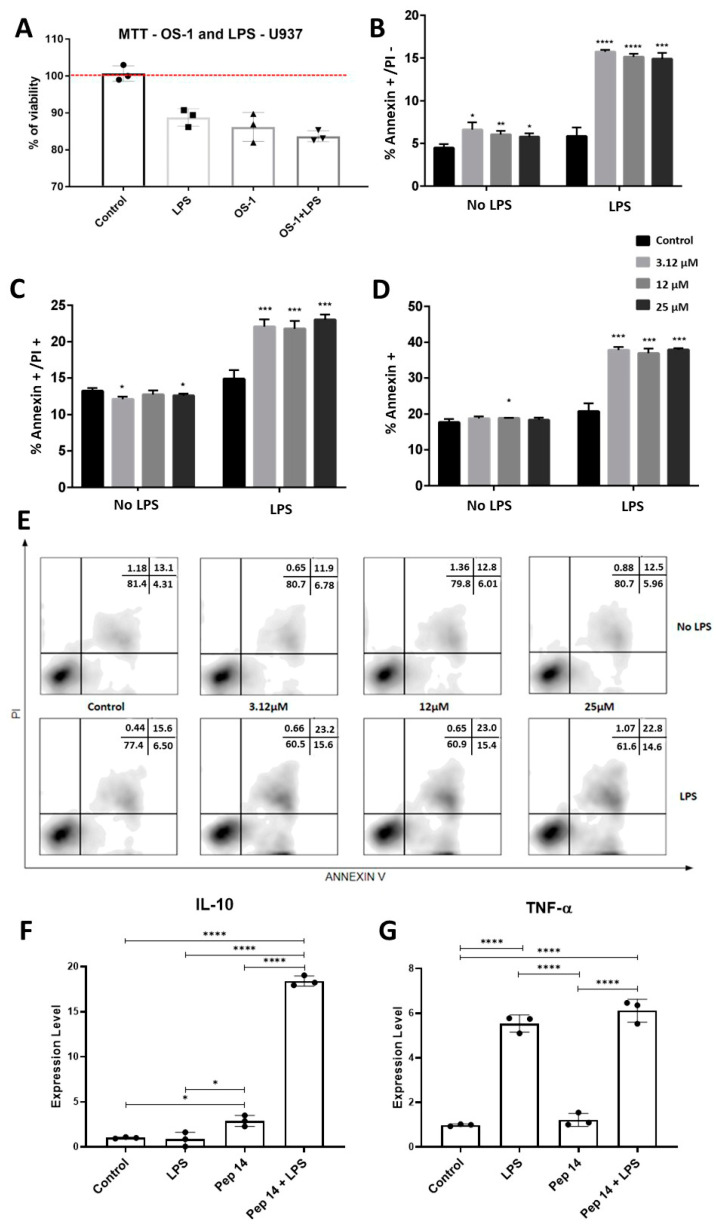
Evaluation of the toxicity of Peptide 14 (Pep 14) and its influence on cytokine production in U-937 cells. To elucidate the effects of Pep 14 on U-937 monocytes, we investigated its toxicity in resting and lipopolysaccharide (LPS)-stimulated cells using the MTT assay (**A**). Then, we determined whether our observations were attributable to an increase in cell death or a limitation in cell proliferation by employing annexin and propidium iodide (PI) analysis by flow cytometry. (**B**) Percentage of annexin V+/PI− cells. (**C**) Percentage of annexin V+/PI+ cells. (**D**) Percentage of total annexin V+ cells. (**E**) Representative dot plots of annexin V/PI staining. After cell viability was evaluated, we investigated the mRNA levels of *IL-10* and *TNF-α* cytokines (**F**,**G**) produced by the treated monocytes using qRT-PCR. Statistical analysis was performed using ANOVA, followed by Tukey’s post hoc test for multiple comparisons. * *p* < 0.05, ** *p* < 0.01, *** *p* < 0.001, **** *p* < 0.0001 between groups.

To investigate the effects of Pep 14 on monocytes and macrophages, we determined whether the peptide would be toxic to resting and LPS-stimulated monocytes of the U-937 cell line when used at the same concentration as that used in PBMCs. The results of the MTT assay revealed that LPS and Pep 14 exerted a modest, non-significant reduction in cell viability (Figure 4A). Also, Pep 14 did not significantly influence the modest cytotoxicity effect of LPS on U-937 monocytes. Such findings corroborate previous observations that LPS promotes monocyte death in a dose-dependent manner, as a result of the stimulation of excessive reactive species production [19]. Nevertheless, considering the rapid cell proliferation observed in U-937, we decided to investigate whether our observations in treated groups might be a result of increased cell death or limited cell proliferation. To do so, we performed annexin V/PI staining in the treated cells, using three different concentrations of Pep 14. Once again, resting and LPS-stimulated cells were investigated. The results demonstrated that the peptide selectively induced apoptosis in LPS-stimulated monocytes in a dose-independent manner (Figure 4B–E).

After cell viability was evaluated, the mRNA levels of cytokines produced by treated monocytes were evaluated using gene expression analysis (Figure 4F,G). The results obtained indicated that Pep 14 significantly induced *IL-10* expression in resting and LPS-stimulated U-937 cells (Figure 4F). In the cells treated with LPS only, *TNF-α* expression was significantly increased (Figure 4G).

### 3.3. Pep14 Prevents LPS-Induced Activation and Promotes Regenerative M2 Polarization in U-937-Derived Macrophages

**Figure 5:**
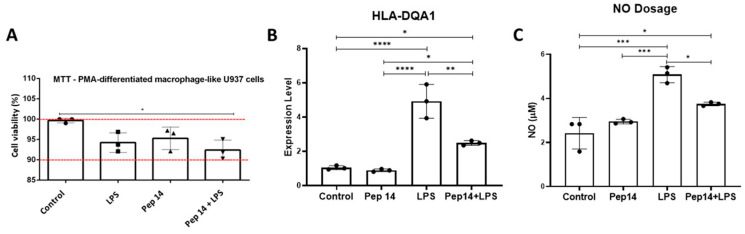
Effects of peptides on U-937 macrophages. The cytotoxicity of Pep 14 and lipopolysaccharide (LPS) was determined using the MTT assay (**A**). Macrophage activation after treatment with Pep 14 and LPS was investigated by analyzing *HLA-DQA1* mRNA expression (**B**) and nitric oxide (NO) production (**C**). Statistical analysis was performed using ANOVA, followed by Tukey’s post hoc test for multiple comparisons. * *p* < 0.05, ** *p* < 0.01, *** *p* < 0.001, **** *p* < 0.0001 between groups.

After the effect of Pep 14 on U-937 monocytes was evaluated, the effect of the peptide on U-937 macrophages was also evaluated. The monocytes were differentiated into macrophages using PMA. Then, Pep 14 and LPS cytotoxicity were evaluated using the MTT assay. The group stimulated with LPS and treated with the peptide showed statistically increased levels of cell death after LPS stimulation compared to the control group (Figure 5A). Nevertheless, such findings were considered as biologically irrelevant since cell viability was greater than 90% in all experimental groups.

Macrophage activation following Pep 14 and LPS treatments was also assessed by analyzing the expression of *HLA-DQA1* mRNA and NO production (Figure 5B,C). The results indicated that Pep 14 treatment did not promote *HLA-DQA1* expression in resting U-937 macrophages. In LPS-stimulated counterparts, *HLA-DQA1* expression was significantly increased. On the other hand, in cells treated with both LPS and Pep 14, *HLA-DQA1* expression was significantly decreased (Figure 5B). NO production was also unaltered by Pep 14 in resting U-937 macrophages, while being significantly induced in LPS-stimulated counterparts and limited in LPS/Pep 14-treated cells (Figure 5C). Both findings support the notion that Pep14 reduces LPS-induced activation of U-937 macrophages, as would be expected from an immunomodulatory compound.

**Figure 6:**
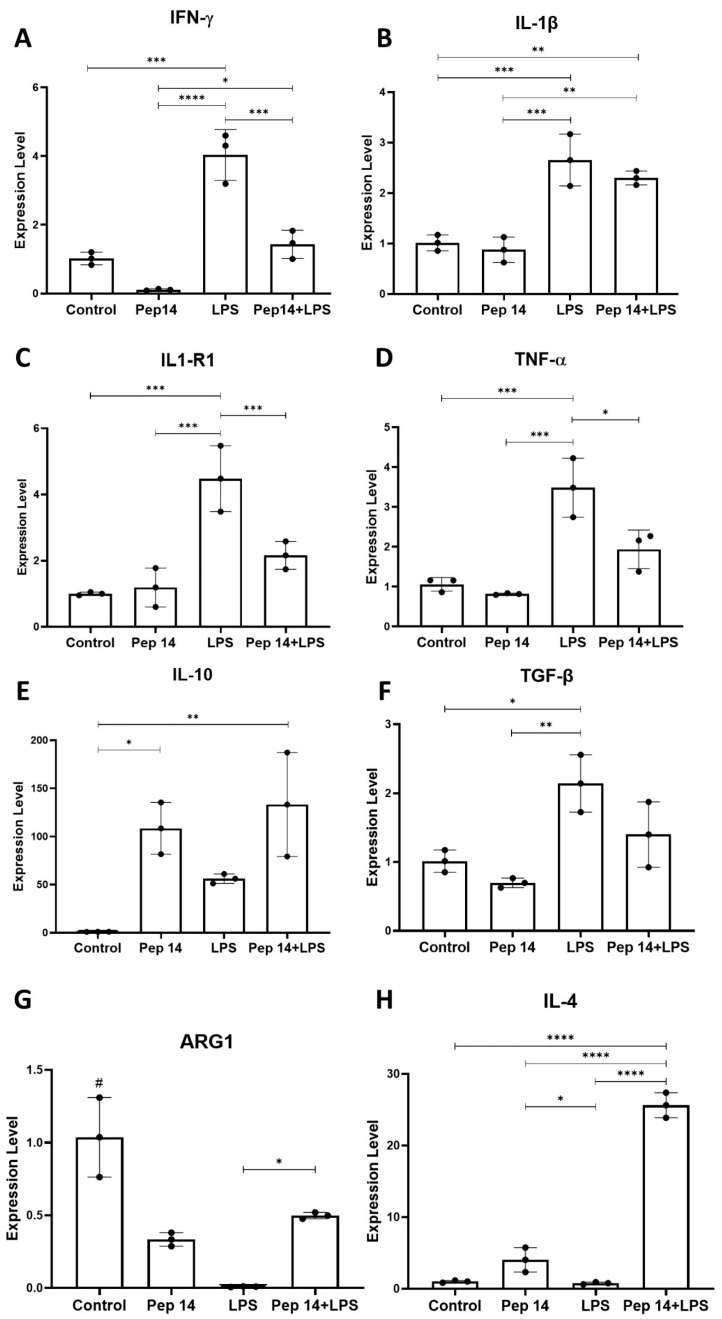
Effects of Pep 14 on macrophage polarization. We evaluated the mRNA expression of the M1 macrophage marker genes *IFN-γ* (**A**), *IL-1β* (**B**), *IL-1R1* (**C**), and *TNF-α* (**D**) in U-937 macrophages. We also determined the mRNA expression levels of the M2 macrophage marker genes *IL-10* (**E**), *TGF-β* (**F**), *ARG1* (**G**), and *IL-4* (**H**). Statistical analysis was performed using ANOVA, followed by Tukey’s post hoc test for multiple comparisons. * *p* < 0.05, ** *p* < 0.01, *** *p* < 0.001, **** *p* < 0.0001 between groups. # *p* < 0.05 between control and experimental groups.

Subsequently, the effects of Pep 14 in macrophage polarization were investigated through qRT-PCR (Figure 6). Our findings show that in resting cells, Pep 14 treatment significantly limited *IFN-γ* mRNA expression (Figure 6A), compared to the untreated control. As expected, LPS-treated cells exhibited a significant increase in the expression of all M1 subset markers, compared to the control group (I*FN-γ*, *IL-1β*, *IL-1R1*, and *TNF-α*) (Figure 6A–D). In LPS/Pep 14-treated cells, it was possible to observe an attenuation of the effect of LPS, resulting in a significant reduction in the expression of *IL-1R1*, *IFN-γ*, and *TNF-α* genes, compared to the LPS-treated group. This mRNA profile indicates an attenuation of the LPS-induced expression of genes associated with the inflammatory M1 macrophage subset.

Next, we examined the genes associated with M2 polarization. In this analysis, the *IL-10*, *TGF-β*, *ARG1*, and *IL-4* transcripts were considered (Figure 6E–H). The results indicated that when the cells were exclusively treated with Pep 14, a significant increase in the expression of *IL-10* compared to the control group could be observed (Figure 6E). Furthermore, while LPS significantly induced *TGF-β* and showed a strong trend towards increased *IL-10* expression (*p* = 0.056) (Figure 6F), it limited *ARG1* expression (Figure 6G) and did not significantly influence *IL-4* (Figure 6H). Pep 14 treatment of LPS-stimulated cells resulted in significantly increased *ARG1* and *IL-4* mRNA levels, in addition to a trend towards increased *IL-10* compared to the LPS-treated group. These findings suggest that, while limiting LPS-induced activation and expression of M1 polarization genes, Pep 14 promoted an M2 mRNA signature in LPS-stimulated macrophages of the U-937 cell line.

**Figure 7:**
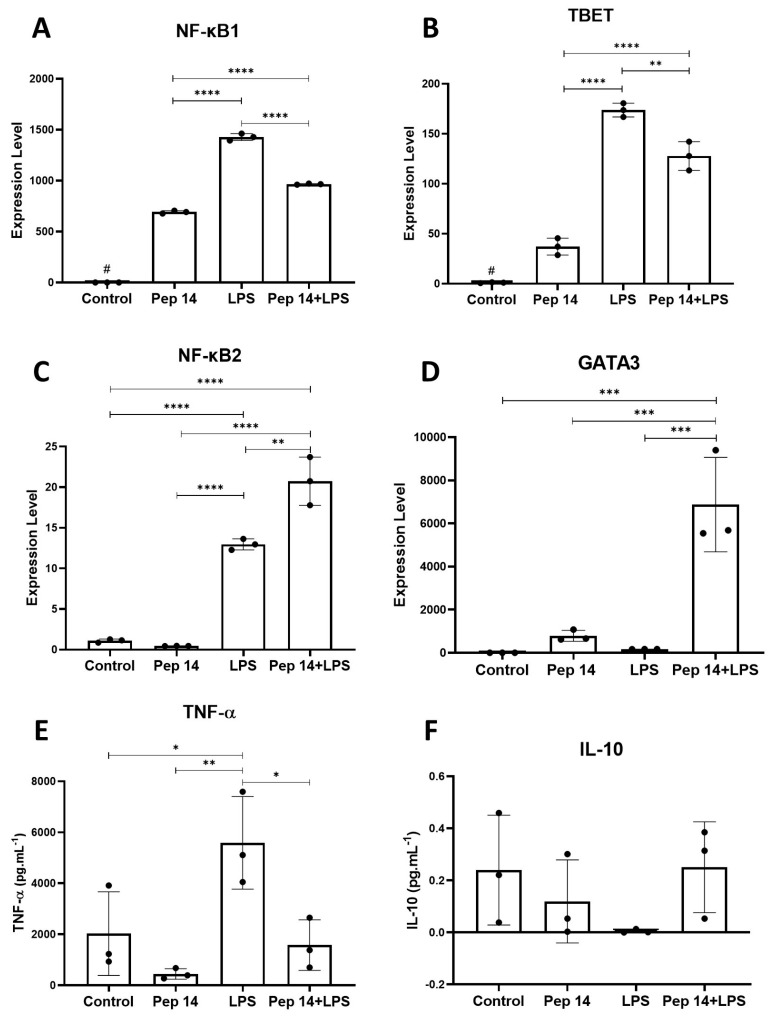
Effects of Pep 14 on macrophage polarization. For a more comprehensive understanding of macrophage differentiation, the *NF-κB1* (**A**), *TBET* (**B**), *NF-κB2* (**C**), and *GATA3* (**D**) gene transcripts were investigated. Next, to validate the data obtained by qRT-PCR, the concentrations of the pro- and anti-inflammatory cytokines TNF-α (**E**) and IL-10 (**F**) in the supernatant of U-937 macrophages were quantified by ELISA. Statistical analysis was performed using ANOVA, followed by Tukey’s post hoc test for multiple comparisons. * *p* < 0.05, ** *p* < 0.01, *** *p* < 0.001, **** *p* < 0.0001 between groups. # *p* < 0.05 between control and experimental groups.

To better understand macrophage differentiation, transcripts belonging to the NF-κB, TBET (M1-related), and GATA3 (M2-related) families were analyzed. Our data revealed that Pep 14 promoted *NF-κB1* and *TBET* expression in resting U-937 macrophages, while LPS treatment induced *NF-κB1*, *TBET*, and *NF-κB2* expression (Figure 7A–C). In LPS-stimulated macrophages treated with Pep 14, the peptide limited the LPS- induced *NF-κB1* and *TBE*T mRNA levels (Figure 7A,B), while significantly increasing the anti-inflammatory *NF-κB2* and M2-related transcript *GATA3* when compared to LPS-treated samples (Figure 7C,D).

To confirm the qRT-PCR data, pro- and anti-inflammatory cytokines were quantified in the supernatant of U-937 macrophages treated with LPS and/or Pep 14, using ELISA. In the absence of LPS stimulation, the peptide failed to significantly influence TNF-α and IL-10 release. Nevertheless, in LPS-stimulated cells, Pep 14 treatment limited the LPS-induced TNF-α release (Figure 7E), while showing a trend towards increased IL-10 release (Figure 7F). Once again, it is possible to suggest that Pep 14 limits M1 cytokine release in LPS-stimulated cells, possibly favoring the M2 macrophage subset profile.

### 3.4. The Secretome of LPS- and Pep 14-Treated Macrophages Promotes Fibroblast Migration and Is Compatible with Functional M2 Macrophage Differentiation

**Figure 8:**
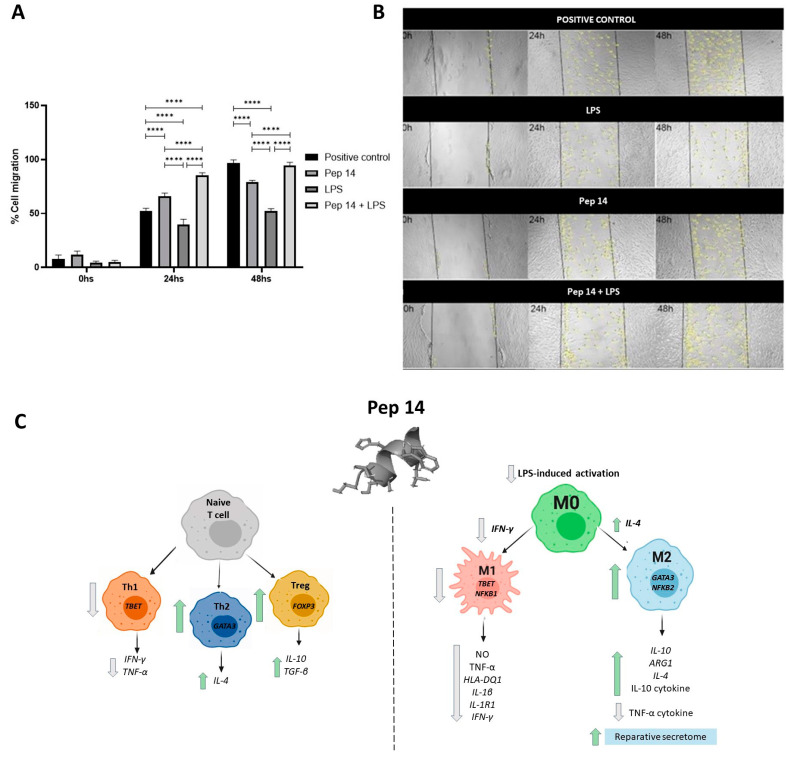
Cell migration assay of fibroblasts treated with macrophage-conditioned medium. (**A**) Analysis of cell migration expressed as a percentage of the control. Statistical analysis was performed using ANOVA, followed by Tukey’s post hoc test for multiple comparisons. **** *p* < 0.0001. (**B**) Representative images of the migration assay. (**C**) Pep 14 exerts direct effects on immune cells, supporting Th2 and Treg subsets in T cells and limiting LPS-driven activation of macrophages, favoring an M2 phenotype. In both PBMCs and macrophages, Pep 14 consistently promoted IL-10 mRNA expression and cytokine release.

While M1 macrophages are generally considered proinflammatory cells, M2 counterparts are associated with an anti-inflammatory/resolving profile [20] that interacts with and influences fibroblast behavior, such as migration [21,22]. In order to confirm that Pep 14 treatment supports functional M2 macrophage polarization following LPS treatment, U-937 macrophages were stimulated with LPS and treated with Pep 14. Then, the cell culture supernatant was collected and used to stimulate human primary dermal fibroblasts in a wound scratch test. While Pep 14 treatment of fibroblasts fails to directly promote fibroblast migration, our analyses revealed that the conditioned media from both resting and LPS-stimulated macrophages treated with Pep 14 significantly promoted fibroblast migration, compared to the conditioned media derived from LPS-treated macrophages (Figure 8).

## 4. Discussion

Immunosenescence contributes to the establishment of a chronic state characterized by exacerbated inflammatory signaling and compromised adaptive immunity [1]. This phenomenon has been implicated in causing and/or aggravating multiple degenerative conditions, such as renal [23], cardiovascular [24], and neurodegenerative diseases [25]. Recently, Pep 14 was identified as a new molecule with senotherapeutic potential, capable of effectively modulating cellular senescence and preventing inflammatory signaling promoted by SASP. Pep 14 was shown to act through the stabilization of PP2A [7], a complex phosphatase comprising heterotrimeric Ser/Thr subunits that regulate several cellular processes. Current evidence suggests that PP2A plays a significant role in inflammatory responses, with alterations being associated with autoimmune diseases [9]. Considering the potential role of this peptide in modulating signaling pathways critical for the regulation of inflammation, we evaluated how immune cells respond to Pep 14.

Under the concentrations tested, Pep 14 was not toxic to resting PBMCs, nor to resting monocytes and macrophages from the U-937 cell line. These results corroborate previous findings from our group showing a lack of toxicity of the peptide in different cell types [7,8]. While the peptide failed to significantly influence T cell activation and proliferation following stimulation with PHA, it promoted the expression of GATA3, FOXP3, and RELB mRNAs, important transcription factors related to T cell differentiation into Th2 and Treg subsets. Simultaneously, the peptide prevented PHA-induced expression of TBET, a gene that controls Th1 differentiation. Furthermore, Pep 14 treatment inhibited PHA-induced IFN-γ expression, while promoting TGF-β, IL-4, and IL-10 mRNA expression. These findings were partially confirmed at the protein level, as we observed significantly increased IL-10 and significantly decreased TNF-a levels in the supernatant of Pep 14-treated cells. Therefore, the results indicate that Pep 14 suppresses Th1-related mRNA expression and the release of cytokines associated with this immune response, while favoring Treg and Th2 phenotypes. Also of note, the effects of Pep 14 treatment differ from those observed for rapamycin, suggesting distinct mechanisms of action. In the case of Pep 14, our observations are in accordance with previous findings showing that the inhibition of PP2A with the small-molecule compound LB100 resulted in an increase in Th1 differentiation in mice [26].

Innate immune cells such as macrophages also change their profile during aging and become relevant drivers of inflammaging and immune diseases [3]. Therefore, favoring an anti-inflammatory phenotype in these cells would be potentially beneficial in delaying such age-related complications. To further explore the potential activities of Pep 14, experiments were carried out using cells of the macrophage model U-937 lineage with the aim of understanding the nature of the response that treatment with this peptide could trigger in monocytes. Cell viability tests revealed that the peptide selectively induced apoptosis in LPS-activated monocytes. It is important to highlight that in inflammatory diseases, the ability to induce apoptosis in activated monocytes is sometimes crucial to control the accumulation of these cells in inflammatory lesions, which can result in inflammation-related tissue damage and remission of the inflammatory disease [27]. Furthermore, numerous studies have suggested that defects in apoptosis are fundamental abnormalities in the pathogenesis of such diseases, and can also have cancer-inducing effects [28,29]. Gene expression analysis revealed that Pep 14 significantly increased IL-10 expression in the resting and LPS-stimulated U-937 cells. Together, these results strongly suggest the anti-inflammatory potential of this peptide in monocytes [30,31].

This anti-inflammatory potential of Pep 14 was also evaluated in U-937-derived macrophages. Again, selective death of cells activated by LPS was observed following peptide treatment, which is potentially relevant in the context of the treatment of inflammatory diseases [32]. Macrophage activation after treatment with Pep 14 and LPS was evaluated by analyzing HLA-DQA1 mRNA expression and NO production, and the results showed that the peptide significantly reduced both of these macrophage activation markers. These results suggest that Pep 14 prevents excessive LPS-induced activation of U-937 macrophages, indicating an inhibitory effect on the LPS-triggered inflammatory response.

Next, we investigated the effects of Pep 14 on macrophage polarization. The results revealed that Pep 14 significantly limited IFN-γ expression and boosted IL-10 expression in resting cells. LPS-treated cells showed a marked increase in the expression of M1 subset markers. In contrast, cells stimulated with LPS and treated with Pep 14 presented a significant increase in ARG1 and IL-4 (M2 markers) mRNA levels, as well as a trend towards increased IL-10 expression, suggesting a Pep14-induced M2 mRNA signature in LPS-stimulated macrophages. This observation was also supported by the analysis of the NF-κB2 and GATA3 transcripts. Concurrently, M1 polarization genes IFN-γ and TNF-α were suppressed in these samples. The reduced expression of M1 polarization genes suggests that Pep 14 treatment may help in preventing macrophages from generating M1 counterparts, while favoring M2 polarization. Given that M2 macrophages and their markers play crucial roles in the resolution of inflammation, wound regeneration, and tissue repair [33,34,35], such observations underscore the possible effect of the peptide in contributing to anti-inflammatory response and tissue regenerative activities.

In order to confirm the M2 polarization profile of Pep 14-treated macrophages, a functional assay was carried out by treating human primary dermal fibroblasts with the conditioned media of treated macrophages. Consistent with the expected regenerative profile of M2 macrophages and their capacity to induce fibroblast cell migration and proliferation [36], the treated fibroblasts presented higher migration rates compared to the experimental controls.

Prior studies have determined that senotherapeutic molecules might impact immune system function, similar to common regulators of senescence and immunity. This is the case of rapamycin, an mTOR (mammalian target of rapamycin) inhibitor, initially described as an antifungal molecule, that was later found to effectively limit T cell proliferation and is currently used in clinical settings as an immunosuppressant medication [37]. The role of mTOR in aging has been consistently shown throughout evolution as a conserved signaling molecule that integrates diverse environmental and intracellular signals. This has led to the establishment of a growing body of evidence regarding the efficacy of rapamycin in increasing lifespan and/or healthspan in the most diverse experimental models, from yeast [38] to monkeys [39]. The effect of rapamycin on immunosenescence is currently under investigation in preclinical [40] and clinical settings [41].

Here, we have used rapamycin as a reference senotherapeutic molecule with established immunosuppressant capacity. Pep 14 presented different immunomodulatory effects compared to rapamycin, likely as a result of the different molecular targets of each compound. While rapamycin effectively limited T cell proliferation, suppressed the mRNA expression of the Th1-regulator TBET, and increased the mRNA expression of TGF-β and IL-4, Pep 14 failed to modulate T cell proliferation but clearly supported Treg differentiation (by promoting FOXP3 mRNA expression) and function (by promoting IL-10 mRNA expression and cytokine release).

Our findings are compatible with the already described capacity of Pep 14 to promote PP2A stabilization. The role of PP2A in the regulation of immune cells was first demonstrated by Taffs et al. [42]. Since then, it has been established that PP2A regulates different signaling pathways, including NF-κB [43], phosphatidylinositol-3-kinase (PI3K)/AKT/mTOR [44], and mitogen-activated protein kinase (MAPK) [45]. In T cells, this is reflected in the suppression of the NF-κB pathway during TCR signaling [43], the inhibition of Th1 differentiation through mTORC1 suppression [26], and the promotion of Th2 and Treg T cell subsets by maintaining mTORC2 and PI3K/AKT pathway function in T cells (leading to Th2 differentiation) and by potentiating IL-2R signaling (leading to Treg differentiation and function) [46]. Additional details of how PP2A modulation impacts T cell function can be found in the recent review by Roy and Batra [47]. In macrophages, PP2A inhibition has been shown to drive M1 polarization [48], possibly through STAT and NF-κB signaling modulation. Importantly, Fingolimod (FTY720, Gilenya) constitutes an example of an immunosuppressant drug that activates PP2A and is currently approved by the FDA to treat multiple sclerosis. Similar to our findings, the drug has been shown to limit inflammation by modulating T cell and macrophage function [49]. Further investigation is necessary to confirm whether PP2A stabilization is the main mechanism related to the effects of Pep 14 described in this study and also the druggability of this compound.

## 5. Conclusions

Taken together, our results show that Pep 14 exerts direct effects on immune cells. In T cells, Pep 14 supports Th2 and Treg subsets, while suppressing the Th1 regulator TBET and Th1-signature cytokines; in macrophages, the peptide limits LPS-induced activation and supports functional M2 cells, while suppressing inflammatory NF-κB1 signaling and M1-signature markers and cytokines. In all cell types, the peptide consistently promoted IL-10 mRNA expression and cytokine release. Further studies are required to uncover the underlying mechanisms and to validate our findings, but the current data support direct immunomodulatory potential beyond the already described senotherapeutic effect of Pep 14.

## Figures and Tables

**Table 1:** Primer sequence.

Transcript	Primer Forward (5′–3′)	Primer Reverse (5′–3′)	T (°C)
ARG1	ATTGAGAAAGGCTGGTCTGC	CATTAGGGATGTCAGCAAAGG	60
FOXP3	TCATCCGCTGGGCCATCCTG	GTGGAAACCTCACTTCTTGGTC	58
GAPDH	ACATCGCTCAGACACCATG	TGTAGTTGAGGTCAATGAAGGG	60
GATA3	AGCCTGTCCTTTGGACCACA	CATCGAGCAGGGCTCTAACC	60
HLA-DQ1	CTCTGTTCCGCAGATTTAGAAGA	GGAGACTTGGAAAACACTGTGACC	60
IFN-γ	ACTGTCGCCAGCAGCTAAAA	TATTGCAGGCAGGACAACCA	60
IL-10	GGCACCCAGTCTGAGAACAG	ACTCTGCTGAAGGCATCTCG	60
IL-1R1	CCTCCAGGATTCATCAACAC	AAAACTCCATATAAGGGCACAC	60
IL1β	ATGATGGCTTATTACAGTGGCAA	GTCGGAGATTCGTAGCTGGA	60
IL-4	TTGTCCACGGACACAAGTGC	TGTTACGGTCAACTCGGTGC	60
NF-κB1	GTGGTGCCTCACTGCTAACT	GGATGCACTTCAGCTTCTGT	58
NF-κB2	TAGCCACAGAGATGGAGGAG	CCGAGTCGCTATCAGAGGT	60
REL	AAAGACTGCAGAGACGGCT	CTCACCACATTGAGGTCACA	58
RELA	GCACAGATACCACCAAGACC	TCAGCCTCATAGAAGCCATC	58
RELB	CATTGAGCGGAAGATTCAAC	GCAGCTCTGATGTGTTTGTG	56
TBET	CAACACGCATATCTTTACTTTCCA	AGCTGAGTAATCTCGGCATTCTG	65
TGF- β1	GCTGTATTTAAGGACACCGTGC	TGACACAGAGATCCGCAGTC	60
TNF-α	CACAGTGAAGTGCTGGCAAC	GATCAAAGCTGTAGGCCCCA	58

## Data Availability

The raw data supporting the conclusions of this article will be made available by the authors on request.

## References

[1] Rodrigues L.P., Teixeira V.R., Alencar-Silva T., Simonassi-Paiva B., Pereira R.W., Pogue R., Carvalho J.L. (2021). Hallmarks of aging and immunosenescence: Connecting the dots. Cytokine Growth Factor Rev..

[2] Pawelec G. (2012). Hallmarks of human “immunosenescence”: Adaptation or dysregulation?. Immun. Ageing.

[3] Guimarães G.R., Almeida P.P., de Oliveira Santos L., Rodrigues L.P., de Carvalho J.L., Boroni M. (2021). Hallmarks of Aging in Macrophages: Consequences to Skin Inflammaging. Cells.

[4] Wikby A., Nilsson B.-O., Forsey R., Thompson J., Strindhall J., Löfgren S., Ernerudh J., Pawelec G., Ferguson F., Johansson B. (2006). The immune risk phenotype is associated with IL-6 in the terminal decline stage: Findings from the Swedish NONA immune longitudinal study of very late life functioning. Mech. Ageing Dev..

[5] Schmitt V., Rink L., Uciechowski P. (2013). The Th17/Treg balance is disturbed during aging. Exp. Gerontol..

[6] Bruunsgaard H., Skinhøj P., Pedersen A.N., Schroll M., Pedersen B.K. (2008). Ageing, tumour necrosis factor-alpha (TNF-*α*) and atherosclerosis. Clin. Exp. Immunol..

[7] Zonari A., Brace L.E., Alencar-Silva T., Porto W.F., Foyt D., Guiang M., Cruz E.A.O., Franco O.L., Oliveira C.R., Boroni M. (2022). In vitro and in vivo toxicity assessment of the senotherapeutic Peptide 14. Toxicol. Rep..

[8] Zonari A., Brace L.E., Al-Katib K., Porto W.F., Foyt D., Guiang M., Cruz E.A.O., Marshall B., Gentz M., Guimarães G.R. (2023). Senotherapeutic peptide treatment reduces biological age and senescence burden in human skin models. NPJ Aging.

[9] Khan M.M., Kalim U.U., Khan M.H., Lahesmaa R. (2022). PP2A and Its Inhibitors in Helper T-Cell Differentiation and Autoimmunity. Front. Immunol..

[10] Serejo T.R.T., Silva-Carvalho A.É., Braga L.D.D.C.F., Neves F.D.A.R., Pereira R.W., Carvalho J.L.D., Saldanha-Araujo F. (2019). Assessment of the Immunosuppressive Potential of INF-γ Licensed Adipose Mesenchymal Stem Cells, Their Secretome and Extracellular Vesicles. Cells.

[11] Pinto S.M., Kim H., Subbannayya Y., Giambelluca M.S., Bösl K., Ryan L., Sharma A., Kandasamy R.K. (2021). Comparative Proteomic Analysis Reveals Varying Impact on Immune Responses in Phorbol 12-Myristate-13-Acetate-Mediated THP-1 Monocyte-to-Macrophage Differentiation. Front. Immunol..

[12] Green L.C., Wagner D.A., Glogowski J., Skipper P.L., Wishnok J.S., Tannenbaum S.R. (1982). Analysis of nitrate, nitrite, and [15N] nitrate in biological fluids. Anal. Biochem..

[13] Nolan T., Hands R.E., Bustin S.A. (2006). Quantification of mRNA using real-time RT-PCR. Nat Protoc..

[14] Liang C.-C., Park A.Y., Guan J.-L. (2007). In vitro scratch assay: A convenient and inexpensive method for analysis of cell migration in vitro. Nat. Protoc..

[15] Terada N., Lucas J.J., Szepesi A., Franklin R.A., Domenico J., Gelfand E.W. (1993). Rapamycin blocks cell cycle progression of activated T cells prior to events characteristic of the middle to late G_1_ phase of the cycle. J. Cell Physiol..

[16] Oh H., Ghosh S. (2013). NF-κB: Roles and regulation in different CD4(+) T-cell subsets. Immunol. Rev..

[17] Hövelmeyer N., Schmidt-Supprian M., Ohnmacht C. (2022). NF-κB in control of regulatory T cell development, identity, and function. J. Mol. Med..

[18] Lee J., Lozano-Ruiz B., Yang F.M., Fan D.D., Shen L., González-Navajas J.M. (2021). The Multifaceted Role of Th1, Th9, and Th17 Cells in Immune Checkpoint Inhibition Therapy. Front. Immunol..

[19] Franceschelli S., Pesce M., Ferrone A., De Lutiis M.A., Patruno A., Grilli A., Felaco M., Speranza L. (2014). Astaxanth in treatment confers protection against oxidative stress in U937 cells stimulated with lipopolysaccharide reducing O_2_^−^ production. PLoS ONE.

[20] Viola A., Munari F., Sánchez-Rodríguez R., Scolaro T., Castegna A. (2019). The Metabolic Signature of Macrophage Responses. Front. Immunol..

[21] Huang J., Chen Y., Zhou L., Ren J., Tian M., Yang Q., Wang L., Wu Y., Wen J., Yang Q. (2024). M2a macrophages regulate fibrosis and affect the outcome after stroke via PU.1/mTOR pathway in fibroblasts. Neurochem. Int..

[22] Li Z., Bratlie K.M. (2021). Fibroblasts treated with macrophage conditioned medium results in phenotypic shifts and changes in collagen organization. Mater. Sci. Eng. C.

[23] Salimi S., Shardell M.D., Seliger S.L., Bandinelli S., Guralnik J.M., Ferrucci L. (2018). Inflammation and Trajectory of Renal Function in Community-Dwelling Older Adults. J. Am. Geriatr. Soc..

[24] Ferrucci L., Fabbri E. (2018). Inflammageing: Chronic inflammation in ageing, cardiovascular disease, and frailty. Nat. Rev. Cardiol..

[25] Xie J., Van Hoecke L., Vandenbroucke R.E. (2022). The Impact of Systemic Inflammation on Alzheimer’s Disease Pathology. Front. Immunol..

[26] Ho W.S., Wang H., Maggio D., Kovach J.S., Zhang Q., Song Q., Marincola F.M., Heiss J.D., Gilbert M.R., Lu R. (2018). Pharmacologic inhibition of protein phosphatase-2A achieves durable immune-mediated antitumor activity when combined with PD-1 blockade. Nat. Commun..

[27] Mangan D.F., Mergenhagen S.E., Wahl S.M. (1993). Apoptosis in human monocytes: Possible role in chronic inflammatory diseases. J. Periodontol..

[28] Boirivant M., Marini M., Di Felice G., Pronio A.M., Montesani C., Tersigni R., Strober W. (1999). Lamina propria T cells in Crohn’s disease and other gastrointestinal inflammation show defective CD2 pathway-induced apoptosis. Gastroenterology.

[29] Ina K., Itoh J., Fukushima K., Kusugami K., Yamaguchi T., Kyokane K., Imada A., Binion D.G., Musso A., West G.A. (1999). Resistance of Crohn’s disease T cells to multiple apoptotic signals is associated with a Bcl-2/Bax mucosal imbalance. J. Immunol..

[30] Boumaza A., Gay L., Mezouar S., Bestion E., Diallo A.B., Michel M., Desnues B., Raoult D., La Scola B., Halfon P. (2021). Monocytes and Macrophages, Targets of Severe Acute Respiratory Syndrome Coronavirus 2: The Clue for Coronavirus Disease 2019 Immunoparalysis. J. Infect. Dis..

[31] Zheng W., Song H., Luo Z., Wu H., Chen L., Wang Y., Cui H., Zhang Y., Wang B., Li W. (2021). Acetylcholine ameliorates colitis by promoting IL-10 secretion of monocytic myeloid-derived suppressor cells through the nAChR/ERK pathway. Proc. Natl. Acad. Sci. USA.

[32] Hortelano S., Zeini M., Castrillo A., Alvarez A.M., Boscá L. (2002). Induction of apoptosis by nitric oxide in macrophages is independent of apoptotic volume decreas. Cell Death Differ..

[33] Hao J., Hu Y., Li Y., Zhou Q., Lv X. (2017). Involvement of JNK signaling in IL4-induced M2 macrophage polarization. Exp. Cell Res..

[34] Nair M.G., Guild K.J., Artis D. (2006). Novel Effector Molecules in Type 2 Inflammation: Lessons Drawn from Helminth Infection and Allergy. J. Immunol..

[35] Ji J., Shu D., Zheng M., Wang J., Luo C., Wang Y., Guo F., Zou X., Lv X., Li Y. (2016). Microbial metabolite butyrate facilitates M2 macrophage polarization and function. Sci. Rep..

[36] Ploeger D.T., Hosper N.A., Schipper M., Koerts J.A., de Rond S., Bank R.A. (2013). Cell plasticity in wound healing: Paracrine factors of M1/M2 polarized macrophages influence the phenotypical state of dermal fibroblasts. Cell Commun. Signal..

[37] Weichhart T. (2018). mTOR as Regulator of Lifespan, Aging, and Cellular Senescence: A Mini-Review. Gerontology.

[38] Yalcin G., Kim J., Seo D., Lee C.K. (2023). FPR1 is essential for rapamycin-induced lifespan extension in *Saccharomyces cerevisiae*. Biochem. Biophys. Res. Commun..

[39] Ross C., Salmon A., Strong R., Fernandez E., Javors M., Richardson A., Tardif S. (2015). Metabolic consequences of long-term rapamycin exposure on common marmoset monkeys (*Callithrix jacchus*). Aging.

[40] Phillips E.J., Simons M.J.P. (2023). Rapamycin not dietary restriction improves resilience against pathogens: A meta-analysis. GeroScience.

[41] Mannick J.B., Teo G., Bernardo P., Quinn D., Russell K., Klickstein L., Marshall W., Shergill S. (2021). Targeting the biology of ageing with mTOR inhibitors to improve immune function in older adults: Phase 2b and phase 3 randomised trials. Lancet Health Longev..

[42] Taffs R.E., Redegeld F.A., Sitkovsky M.V. (1991). Modulation of cytolytic T lymphocyte functions by an inhibitor of serine/threonine phosphatase, okadaic acid. Enhancement of cytolytic T lymphocyte-mediated cytotoxicity. J. Immunol..

[43] Eitelhuber A.C., Warth S., Schimmack G., Düwel M., Hadian K., Demski K., Beisker W., Shinohara H., Kurosaki T., Heissmeyer V. (2011). Dephosphorylation of Carma1 by PP2A negatively regulates T-cell activation. EMBO J..

[44] Peterson R.T., Desai B.N., Hardwick J.S., Schreiber S.L. (1999). Protein phosphatase 2A interacts with the 70-kDa S6 kinase and is activated by inhibition of FKBP12–rapamycin associated protein. Proc. Natl. Acad. Sci. USA.

[45] Chung H., Brautigan D.L. (1999). Protein phosphatase 2A suppresses MAP kinase signalling and ectopic protein expression. Cell Signal..

[46] Ding Y., Yu A., Tsokos G.C., Malek T.R. (2019). CD25 and Protein Phosphatase 2A Cooperate to Enhance IL-2R Signaling in Human Regulatory T Cells. J. Immunol..

[47] Roy S., Batra L. (2023). Protein Phosphatase 2A: Role in T Cells and Diseases. J. Immunol. Res..

[48] Mondal I., Das O., Lu R., Ho W. (2020). Protein Phosphatase 2A Inhibition in Macrophages Enhanced Type I Interferon Production and M1 Polarization. Neurosurgery.

[49] Pournajaf S., Dargahi L., Javan M., Pourgholami M.H. (2022). Molecular Pharmacology and Novel Potential Therapeutic Applications of Fingolimod. Front. Pharmacol..

